# Clinical and Environmental Harms of Quaternary Ammonium Disinfectants and the Promise of Ultraviolet-C (UV-C) Alternatives: A Narrative Review

**DOI:** 10.7759/cureus.84022

**Published:** 2025-05-13

**Authors:** Mitchell K Ng, Michael A Mont, Peter M Bonutti

**Affiliations:** 1 Orthopedic Surgery, Maimonides Medical Center, Brooklyn, USA; 2 Orthopedic Surgery, LifeBridge Health, Baltimore, USA; 3 Orthopedic Surgery, Sarah Bush Lincoln Bonutti Clinic, Effingham, USA

**Keywords:** chemical disinfectants, covid-19, disinfection efficiency, quaternary ammonium compounds, uv-c

## Abstract

Quaternary ammonium compounds (QACs) are widely used disinfectants whose application expanded dramatically during the COVID-19 pandemic, raising concerns about long-term safety and environmental impact. This narrative review begins by outlining the chemical structure, mechanisms of action, and widespread use of QACs across healthcare, consumer, and industrial settings. We examine toxicologic findings from in vitro and animal studies, highlighting mitochondrial dysfunction, oxidative stress, and reproductive toxicity at low-dose exposures. Clinical and public health data are reviewed, linking QAC exposure to respiratory symptoms, dermatitis, and potential reproductive risks among healthcare workers and other vulnerable populations. Environmental harms are also discussed, including QAC persistence in soil and water, ecotoxicity in aquatic species, and potential for bioaccumulation. We then explore ultraviolet-C (UV-C) disinfection as a non-chemical alternative, demonstrating comparable antimicrobial efficacy without the toxicological or ecological drawbacks of QACs. Despite the growing use of QACs, few studies have comprehensively reviewed their adverse effects or evaluated UV-C as a viable substitute. This narrative review fills a critical gap by synthesizing current evidence across disciplines and highlighting the need for safer disinfection practices. We advocate for the broader adoption of UV-C technologies to reduce chemical exposure, protect public health, and promote environmental sustainability.

## Introduction and background

The COVID-19 pandemic catalyzed an unprecedented surge in the global use of chemical disinfectants [1]. As societies sought to curb viral transmission, routine surface cleaning practices became a mainstay in homes, schools, healthcare facilities, and workplaces [2]. Disinfectant products - especially those with quick-drying and broad-spectrum antimicrobial claims - were rapidly adopted as essential tools in infection control [3]. Among the vast array of available agents, quaternary ammonium compounds (QACs) quickly rose to prominence [4]. Their popularity stems from their potent antimicrobial activity, compatibility with various formulations, and relatively low odor. QACs are now found in an array of products: disinfecting wipes, sprays, foggers, and personal care products, many of which are used daily by millions of people [5,6].

However, the increased reliance on QACs has outpaced the scientific scrutiny surrounding their safety, particularly under conditions of frequent, chronic, and widespread use [4,7]. QACs represent a large and chemically diverse class of surfactants with antimicrobial properties, but their systemic and environmental toxicity profiles remain under-investigated. The QAC industry is a $20 billion global market, producing over 700,000 tons annually, posing a major environmental risk. While effective against a range of pathogens, mounting evidence suggests that QACs are associated with a spectrum of biological harms - including mitochondrial dysfunction, reproductive toxicity, and aquatic ecotoxicity [7]. QAC exposure has been associated with a wide range of adverse health effects, including respiratory irritation, occupational asthma, contact dermatitis, reproductive toxicity, developmental neurotoxicity, mitochondrial dysfunction, immunosuppression, and potential links to neurodegenerative and demyelinating diseases [7]. Furthermore, because QACs are cationic and persistent, they tend to accumulate on surfaces and in environmental reservoirs such as dust, water, and sediment, leading to prolonged exposure even after disinfection events [4].

Despite their widespread use, QAC-based disinfectants are not instantaneous in action and require prolonged surface contact to be effective. Most QAC wipes require a continuously wet surface for at least four minutes to achieve disinfection, yet over 50% of surfaces begin to dry within 60 seconds at room temperature [8]. This necessitates re-wiping the same area multiple times - up to four applications - to maintain the required wet contact time. EPA-approved product labeling, however, does not reflect these real-world limitations and often misleads users into believing that a single wipe is sufficient. This raises significant concerns about EPA oversight and its role in ensuring accurate public health guidance [7]. Moreover, there is no practical method for users to visually confirm whether a surface has been adequately disinfected. Compounding these issues, microbial resistance to QACs - including bacteria and viruses - has been increasingly documented, further undermining the long-term efficacy and safety of these agents [6].

Additionally, QAC wipes are only approved for use on hard, non-porous surfaces and explicitly caution against use around food, liquids, or food-contact areas. Labels from brands, such as Clorox and Lysol, also instruct users to wear gloves or wash hands after use - an implicit acknowledgment of potential toxicity [9]. This raises an important question: if surfaces must be wiped with water to remove residues and hands washed after use, why not simply use soap and water in the first place? The toxicity of residues, if ingested or transferred to food, further complicates their use in everyday settings. Are users even aware of these limitations and precautions [6,10]? The lack of public education, combined with misleading labeling, exacerbates unnecessary exposure and reinforces the need for safer, more transparent disinfection strategies.

Given all of these concerns, in this narrative review, we aim to: 1) summarize the expanding body of literature that identifies QAC-related harms, from cellular toxicology to clinical and environmental outcomes; 2) highlight the particular vulnerability of certain populations - such as healthcare workers, custodial staff, children, and pets - who may be exposed to disproportionately high levels of QACs; and 3) introduce ultraviolet-C (UV-C) disinfection as a promising non-chemical alternative that offers effective microbial control without the toxicological burden posed by QACs. UV-C technologies have demonstrated high efficacy against bacterial, viral, and fungal organisms, and unlike QACs, they do not leave chemical residues or contribute to indoor air contamination.

## Review

Methods

This narrative review was informed by a targeted search of peer-reviewed literature, regulatory agency reports, and clinical studies from databases including PubMed and Google Scholar. Search terms included “quaternary ammonium compounds”, “QAC toxicity”, “UV-C disinfection”, “chemical disinfection”, “clinical effect”, and/or “environmental impact”. Articles published between 2010 and 2025 were prioritized, with a focus on studies addressing human health effects, environmental impact, and disinfection efficacy.

Quaternary ammonium disinfectants: definition and uses

QACs are cationic surfactants characterized by a central nitrogen atom bonded to four organic groups, resulting in a permanently charged ammonium ion [9]. This structure disrupts microbial membranes, leading to leakage of cellular contents and eventual cell death [9]. Their mode of action involves electrostatic attraction to negatively charged microbial membranes, insertion of hydrophobic alkyl chains, and subsequent membrane destabilization [11]. These physicochemical properties make QACs potent against bacteria, some viruses, and fungi.

Common subclasses include benzalkonium chlorides (BACs), alkyltrimethylammonium compounds (ATMACs), dialkyldimethylammonium chlorides (DADMACs), and more complex formulations like alkyldimethyl ethylbenzyl ammonium compounds (EBACs) [6]. These agents are integral to numerous consumer and institutional products, including hospital-grade disinfectants (surface wipes, sprays, foggers), household cleaners and personal care items (hand sanitizers, shampoos, deodorants), industrial and agricultural agents (antistatic coatings, herbicides, food production sanitizers), textiles and surface treatments (antimicrobial coatings on fabrics and medical devices), and other (preservatives, mouthwash, nasal sprays) (Table 1) [1,6,7].

**Table 1:** List of commonly used quaternary ammonium compounds

Category	Trade name	Key quaternary ammonium compound(s)
Household cleaners & personal care items	Lysol® disinfecting wipes	Alkyl (C12-C16) dimethyl benzyl ammonium chloride
	Clorox® disinfecting wipes	Alkyl dimethyl benzyl ammonium chloride (ADBAC)
	Wet Ones® antibacterial wipes	Benzalkonium chloride (BAC)
	Suave® hand sanitizer	Benzalkonium chloride (BAC)
Hospital-grade disinfectants	Sani-Cloth® AF3	Didecyl dimethyl ammonium chloride (DDAC), ADBAC
	CaviWipes™	Benzalkonium chloride (BAC), DDAC
	Clorox Healthcare® germicidal wipes	Alkyl dimethyl benzyl ammonium chloride
	Lysol® IC quaternary disinfectant cleaner	Alkyl dimethyl benzyl ammonium chloride
Industrial & agricultural agents	Steramine® sanitizing tablets	Didecyl dimethyl ammonium chloride (DDAC)
	Zep® quat sanitizer	Alkyl dimethyl benzyl ammonium chloride, DDAC
	Maqquat® 225N-80	DDAC, alkyl dimethyl benzyl ammonium chloride
	Adsee® 799 (Solvay)	Tallow trimethyl ammonium chloride
Textiles & surface treatments	Microban® Antimicrobial Additives	Benzalkonium chloride (BAC), DDAC
	Aegis® microbe shield	3-(Trimethoxysilyl)propyldimethyloctadecyl ammonium chloride
	Silvadur™ (DuPont)	Polymeric QACs (proprietary blends)
	BioCote® antimicrobial treatment	Mixtures of QACs or metal ions (QAC-based versions common in healthcare)
Other	Visine eye drops	Benzalkonium chloride (BAK)
	Neutrogena shampoo	Cetrimonium chloride
	Colgate mouthwash	Cetylpyridinium chloride (CPC)

In addition to surface disinfectants, QACs are also found in consumer products such as preservatives, mouthwashes, and nasal sprays-raising questions about regulatory oversight, especially given that the Environmental Protection Agency (EPA) regulates these compounds primarily as disinfectants, while systemic exposure through mucosal routes receives less scrutiny [11]. Recent studies have identified immunotoxic effects of QACs, including decreased production of immunoglobulins IgM and IgG, which may impair immune responses [4,6]. QAC exposure has also been implicated in the disruption of cholesterol homeostasis, a critical regulator of cellular membrane structure and metabolic signaling. Furthermore, resistance development has been well-documented among bacterial and viral species exposed to QAC disinfectants, though data on resistance patterns in molds and fungi remain limited [8]. By contrast, UV-C technologies - such as UVCeed (Bonutti Technologies, Effingham, IL) - have demonstrated robust antifungal efficacy and are widely implemented in HVAC systems for mold and fungal spore reduction, highlighting another critical advantage of non-chemical disinfection strategies. Their use expanded dramatically during the COVID-19 pandemic, with QACs featuring in over 50% of EPA-registered disinfectants effective against SARS-CoV-2 [2,6]. This spike was driven by urgency and convenience, often without full consideration of alternative disinfection modalities or downstream impacts.

Despite their prevalence, QACs remain poorly characterized from a regulatory standpoint. The U.S. EPA groups QACs based on structural similarities established in 1988, which do not account for significant variations in toxicity, persistence, and environmental fate among QAC subclasses [9]. Moreover, labeling regulations do not always require full ingredient disclosure, making it difficult for consumers, clinicians, and occupational health professionals to assess risks and benefits (Table 2) [2,4,9,10].

**Table 2:** Summary of benefits and risks of quaternary ammonium compounds (QACs)

Domain	Benefits	Risks
Antimicrobial efficacy	Broad-spectrum activity against bacteria, viruses, and fungi	Overuse may contribute to antimicrobial resistance (AMR)​
Infection control	Effective surface disinfection in healthcare, schools, and public spaces	Limited real-world data on reduction of infection transmission; potential for false sense of security​
Product versatility	Incorporated into wipes, sprays, foggers, hand sanitizers, and coatings	Ubiquitous presence leads to chronic low-level human and environmental exposure
Regulatory approval	Approved by EPA, FDA, and global agencies for use in specific formulations	Outdated regulatory groupings and limited oversight on cumulative exposure and mixtures​
Surface compatibility	Non-corrosive, pH-neutral, compatible with many surfaces	Leaves persistent chemical residues on surfaces and dust, contributing to prolonged exposure
Human health effects	Low acute toxicity in controlled doses (according to industry-sponsored studies)	Associated with respiratory irritation, contact dermatitis, and potential reproductive toxicity in independent studies​
Environmental impact	Limited volatility reduces airborne spread	Persistent in soil, dust, wastewater, and aquatic systems; bioaccumulation and ecotoxicity documented​
Occupational use	Convenient for routine hospital disinfection	High exposure risk for healthcare and custodial workers, with links to asthma and hypersensitivity​

Toxicologic data: in vitro and animal studies

In vitro studies have provided significant insight into the cellular-level toxicity of QACs. For example, Hrubec et al. demonstrated that BACs and DDACs induce mitochondrial dysfunction in mouse neural and liver cell lines at concentrations as low as 1 µM, disrupting oxygen consumption and ATP production [12]. Similarly, DADMAC exposure was associated with increased reactive oxygen species (ROS) generation, lipid peroxidation, and compromised mitochondrial membrane potential [11]. These effects were observed at concentrations relevant to environmental exposure scenarios, suggesting a potential for chronic bioactivity at low doses [12]. Mechanistic studies confirm that QACs interfere with the mitochondrial electron transport chain and initiate oxidative stress responses through activation of NF-κB and MAPK pathways [13]. Disruption of cell membrane integrity has been directly observed using electron microscopy, confirming QAC-induced micelle formation within phospholipid bilayers [14].

In animal models, repeated QAC exposure has been linked to reproductive and developmental toxicity. Studies by Melin et al. and Hrubec et al. involving chronic dietary QAC exposure in mice revealed reduced fertility, altered estrous cycles, decreased sperm motility, and increased incidence of neural tube defects in fetuses [12,15,16]. Notably, these effects occurred at doses that did not produce overt toxicity in adult animals, indicating a potential for reproductive-specific sensitivity. Further, developmental neurotoxicity was observed in mouse offspring exposed in utero to BAC/DDAC mixtures, with persistent behavioral changes and hippocampal alterations detected in adolescence [16]. Immune system modulation was also noted, including decreased thymus weights and altered cytokine expression profiles [12].

Dose-response relationships remain a key limitation in QAC risk characterization. While some effects occur at micromolar or sub-micromolar concentrations, inconsistencies in formulation, administration route, and mixture composition make extrapolation challenging. Industry-sponsored studies often utilize high-dose thresholds and report only apical endpoints, whereas independent academic investigations highlight low-dose mechanistic effects not captured in traditional toxicology assays [7,9]. Together, these findings indicate a growing need to reassess QAC safety based on modern toxicological principles, incorporating sensitive molecular endpoints, realistic exposure routes, and cumulative risk assessment frameworks. As low-dose bioactivity is increasingly linked to chronic health effects, the precautionary principle should guide regulatory decisions concerning QAC use and alternatives.

Clinical and public health impacts

QACs are associated with a growing range of clinical and public health concerns, particularly in environments with frequent disinfectant use [11]. These impacts manifest across both occupational and community settings, with mounting evidence from epidemiologic investigations, case reports, and controlled exposure studies [12].

In addition to contact-related effects, inhalation of QAC aerosols poses a significant health risk. Several studies have documented allergic responses and hypersensitivity reactions, including occupational asthma and rhinitis, particularly among workers using spray disinfectants in poorly ventilated environments [17-19]. Inhaled QACs can irritate the respiratory epithelium, trigger immune responses, and-due to their lipophilic nature-may be absorbed into the bloodstream via the alveolar-capillary interface [17]. This raises further concern about potential systemic effects following repeated or prolonged respiratory exposure, especially in high-use settings such as hospitals, schools, and public transportation systems [18].

QACs also present significant environmental concerns. These compounds frequently enter the water supply through runoff, wastewater, and improper disposal, subsequently contaminating aquatic ecosystems and even the food supply via uptake in animals and crops [11]. Their environmental breakdown is highly variable, contributing to long-term persistence in soils and sediments. With over 700,000 tons manufactured globally each year, the cumulative environmental burden is substantial and growing, underscoring the urgent need for alternative disinfection methods with lower ecological impact [6].

Occupational exposure

Healthcare workers, janitorial staff, and institutional cleaners represent the most heavily exposed populations [6,11]. Routine use of QAC-containing sprays and wipes has been linked to adverse respiratory and dermatologic outcomes. Notably, occupational asthma has been repeatedly observed in settings with high disinfectant use. Cross-sectional studies and workplace investigations, such as those by Rosenman et al. [19], have implicated QACs - especially BAC and didecyldimethylammonium chloride (DDAC) - in new-onset asthma and exacerbation of pre-existing respiratory conditions [20]. Symptoms typically include wheezing, cough, chest tightness, and reversible airflow obstruction, consistent with irritant-induced or sensitizer-induced asthma phenotypes.

Contact dermatitis is also common, with both irritant and allergic subtypes reported among individuals handling QAC-containing products [21]. Dermatology case series and occupational health surveillance have documented sensitization to specific QAC agents, confirmed by patch testing [22]. Experimental studies from the National Institute for Occupational Safety and Health (NIOSH) demonstrated that DDAC and BAC can trigger mixed-type hypersensitivity responses in murine models, lending biological plausibility to observed human reactions [19,20].

Emerging research raises concern about the potential reproductive effects of chronic occupational exposure. A study by Hrubec et al. found decreased fertility in female laboratory workers regularly handling QACs, paralleling findings from rodent models where prolonged exposure resulted in impaired reproductive success and altered estrous cycling [8,12]. While current data remain limited and causality has not been firmly established, these signals underscore the need for precaution and further investigation in reproductive-age populations.

Community exposure

Outside of occupational settings, QAC exposure is widespread yet diffuse, primarily through contamination of indoor air, surfaces, and settled dust [19]. During and after the COVID-19 pandemic, disinfection practices surged in homes, schools, gyms, and public spaces-leading to persistent QAC residues in indoor environments [20]. Analytical surveys of household dust routinely detect BACs and DADMACs at concentrations that correlate with cleaning frequency [23]. These residues readily transfer to skin, particularly in children who engage in frequent floor play and hand-to-mouth activity, raising concerns about chronic low-dose exposure during critical developmental windows.

Pregnant individuals represent another vulnerable group [14,16]. Although human epidemiologic data are sparse, animal studies consistently demonstrate developmental toxicity following prenatal QAC exposure, including fetal growth restriction and neurodevelopmental alterations [15]. Given these findings, precautionary measures to reduce exposure during pregnancy are advisable until further human studies are conducted.

Domestic pets are also at risk. Dogs and cats may ingest QAC residues through grooming behavior or contact with recently treated surfaces [24]. Veterinary data are currently limited, but case reports suggest potential gastrointestinal and dermatologic irritation, highlighting the need for more systematic studies.

Clinical reports and observational data

A growing body of clinical case reports and observational studies supports the association between QAC exposure and adverse health outcomes. While most evidence is derived from cross-sectional designs or case series-limiting definitive conclusions about causality - the consistency of reported symptoms and mechanistic alignment with in vitro and animal data enhance credibility [19,23]. Recurrent findings across respiratory, dermatologic, and reproductive domains suggest that observed harms are not incidental [13,16,22]. Moreover, the absence of mandatory ingredient labeling for many commercial cleaning products presents a major barrier to accurate exposure assessment and public health surveillance. A recent study by Cohn et al. highlighted potential adverse effects of QACs, which were found to be selectively cytotoxic to developing oligodendrocytes, triggering the integrated stress response and apoptosis, with effects confirmed in both mouse models and human brain organoids [25,26]. Given oligodendrocytes’ key role in myelination and support of neuronal function, their disruption may contribute to neurodevelopmental disorders like autism and demyelinating diseases such as multiple sclerosis [25]. Moreover, QACs are highly lipophilic, enabling them to cross the blood-brain barrier and bind to its tight junctions, where they may induce permanent structural damage and further compromise central nervous system integrity [7].

In sum, the clinical and public health literature increasingly points to the potential for QACs to harm exposed populations, particularly in settings with frequent or prolonged use [5,7,11]. These findings justify calls for improved ingredient transparency, standardized labeling, targeted biomonitoring, and the development of safer disinfection strategies to protect vulnerable populations.

Environmental impact and ecotoxicity

QACs exhibit substantial environmental persistence due to their strong adsorption to soil, sediment, and dust particles [10]. Their positive charge binds tightly to negatively charged surfaces such as clay minerals and organic matter, making them resistant to biodegradation [27]. Wastewater treatment does not effectively remove QACs, leading to their accumulation in biosolids, effluent, and surface waters [28]. Concentrations in sediment cores from lakes and estuaries indicate increasing deposition since the 1950s, with pandemic-associated spikes in urban wastewater streams.

QACs are frequently detected in wastewater effluent: 10-100 µg/L, Surface water: 0.01-1.0 µg/L, Sediment: up to 100 mg/kg, and Biosolids: 10-500 mg/kg dry weight [7,9]. Aquatic toxicity is well-documented. QACs disrupt algal growth, impair reproduction in crustaceans, and induce sublethal effects in fish, including oxidative stress and altered behavior. Some QACs have LC50 values in the low microgram per liter range, with no observed effect concentrations (NOECs) below 1 µg/L for sensitive aquatic species [5].

Mixtures of QACs may exert additive or synergistic toxicity, a critical consideration given that environmental samples often contain multiple QACs. Bioaccumulation has been observed in aquatic invertebrates and plants, though biomagnification up the food chain remains understudied. Pet exposure remains underexplored but is plausible given behavior patterns and shared indoor environments. Chronic low-dose ingestion of QAC residues may pose long-term health risks to companion animals, especially those with dermal or gastrointestinal sensitivities [5,7].

UV-C disinfection: efficacy and safety profile

Ultraviolet (UV) radiation constitutes approximately 10% of the sun’s energy that reaches Earth [29]. In small, controlled amounts, UV exposure can offer benefits such as stimulating vitamin D production and skin tanning. However, excessive exposure can lead to sunburn, skin damage, and ocular injury. Therefore, the clinical use of UV-C light - defined as wavelengths between 200 and 280 nanometers - requires careful shielding and strict dose control to avoid unintended harm to human tissue [29].

UV-C inactivates microorganisms by causing photochemical damage to their DNA and RNA, thereby halting replication and rendering them non-infectious. This non-chemical mechanism avoids many of the limitations and toxicological concerns associated with disinfectants like QACs [30]. UV-C can inactivate a broad spectrum of pathogens, including enveloped and non-enveloped viruses, Gram-positive and Gram-negative bacteria, bacterial spores, and fungal organisms.

Evidence from controlled studies shows that UV-C achieves log-3 to log-6 reductions in bacterial load on hospital surfaces, air systems, and high-touch items like keyboards and bed rails [31,32]. In healthcare settings, UV-C has been used for terminal room disinfection, reducing the incidence of healthcare-associated infections (HAIs) caused by Clostridioides difficile, MRSA, and VRE [33].

Unlike chemical disinfectants, UV-C leaves no toxic residue, does not contribute to antimicrobial resistance, and eliminates concerns of systemic absorption. These systems can also be automated, reducing manual labor and minimizing human error [34].

Safety considerations include shielding to prevent direct human exposure, which can damage skin and eyes. Dosimetry systems must ensure adequate UV dose delivery for effective pathogen inactivation. Integration into workflow requires scheduling disinfection during unoccupied periods or using far-UV systems (<230 nm) with emerging safety profiles [32,35].

Compared to QACs, UV-C offers non-contact, non-consumable disinfection, no chemical waste or indoor air contamination, elimination of chemical exposure for workers and patients, compatibility with smart buildings and automation, and rapid disinfection cycles with validated efficacy (Table 3) [1,11,22,31].

**Table 3:** Comparative advantages of UV-C disinfection over QACs and other chemical disinfectants UV-C: ultraviolet-C, QAC: Quaternary ammonium compound

Feature	UV-C disinfection	QACs/chemical disinfectants
Mechanism of action	Physical: DNA/RNA disruption via photodamage	Chemical: membrane disruption, protein denaturation
Residue	None – no chemical residues left on surfaces	Persistent residues remain on surfaces and dust
Toxicity to humans	None if properly shielded; no systemic absorption	Skin/eye irritation, asthma, and possible reproductive effects
Antimicrobial resistance risk	None – non-chemical mechanism	Documented resistance development in microbes
Environmental impact	No contamination of air, soil, or water	Detected in dust, wastewater, soil, and biota
Disinfection speed	Rapid – seconds to minutes	Slower; often requires dwell time and wiping
Surface coverage	Line-of-sight only; requires strategic placement	Can cover non-visible areas if properly wiped
Air disinfection capability	Yes – via upper-room or HVAC-integrated systems	No – limited to surfaces
Operational cost	Higher upfront, lower long-term (no consumables)	Low upfront, ongoing costs for products and PPE
Ease of automation	Easily automated; programmable cycles	Manual application required
Odor and volatility	Odorless, non-volatile	Many products have strong odors, some volatile

Adoption barriers include capital costs, space constraints, and user training [4-6,9,33]. However, cost-effectiveness improves over time when accounting for reduced infection rates, fewer chemical purchases, and lower waste management expenses.

Technological advances have made UV-C more practical and safer to implement. A notable example is UVCeed [31,33], a next-generation, intelligent disinfection device designed for both efficacy and user safety. UVCeed incorporates the following safety and performance features: Automated Dose Control to regulates UV-C output to deliver effective microbial kill without overexposure, an integrated Camera to provide real-time visual feedback, a proximity sensor to adjusts output based on distance and time and an AI-Based Safety Algorithm that shuts off the device if a human body part, pet, or any unintended object enters the field of view, recalculating the dose as needed to ensure continued safety [36].

## Conclusions

The widespread use of QACs has outpaced scientific understanding of their long-term health and environmental consequences. Evidence from toxicological, clinical, and ecological studies increasingly implicates QACs in a range of adverse effects, from mitochondrial and reproductive toxicity to respiratory illness and environmental persistence. Despite their ubiquity in healthcare and community settings, regulatory oversight and public awareness remain limited. Future research should focus on longitudinal human studies to assess chronic QAC exposure, particularly among vulnerable populations, and clarify mechanisms underlying observed toxicities. Comparative studies evaluating the efficacy, safety, and environmental impact of UV-C versus QACs are also needed. Additionally, enhanced biomonitoring and updated regulatory frameworks are essential to guide safer disinfection practices. UV-C disinfection offers a compelling alternative, providing effective microbial control without chemical residues, systemic toxicity, or ecological harm. This narrative review underscores the need to reassess current disinfection practices and adopt safer, sustainable solutions-particularly in high-risk environments like hospitals, schools, and long-term care facilities. Integrating UV-C into routine protocols may reduce chemical burden, protect vulnerable populations, and align infection control strategies with broader public health and environmental goals.
